# Promoting healthy behaviour with financial incentives: three challenges and solutions for large scale implementation

**DOI:** 10.1007/s10198-025-01893-1

**Published:** 2026-02-03

**Authors:** Stefan A. Lipman, David R. de Buisonjé, Koen van der Swaluw

**Affiliations:** 1 Erasmus School of Health Policy & Management, Erasmus University Rotterdam, Rotterdam, the Netherlands; 2 Erasmus Centre for Health Economics Rotterdam, Erasmus University Rotterdam, Rotterdam, the Netherlands; 3 Department of Behaviour and Health, Centre for Prevention, Lifestyle and Health, National Institute for Public Health and the Environment, Bilthoven, the Netherlands; 4 Human Centered Design, Faculty of Industrial Design, Technical University Delft, Delft, the Netherlands; 5 Health, Medical and Neuropsychology Unit, Institute of Psychology, Leiden University, Leiden, the Netherlands; 6 Department of Economic Policy, Ministry of Health, Welfare and Sport, the Hague, the Netherlands; 7 Department of Economics, Radboud University Nijmegen, Nijmegen, the Netherlands

**Keywords:** Financial incentives, Health behaviour change, Deposit contract, Commitment devices, Tailored incentives, D9, I1

## Abstract

Financial incentives are a promising intervention to promote healthier behaviours and potentially reduce health inequalities. Despite robust evidence supporting their effectiveness in encouraging actions such as smoking cessation, increased physical activity, and improved diet, large-scale implementation of financial incentives in Europe remains limited. This perspective identifies three key challenges impeding their broader use: (1) difficulties in reaching the populations most in need, (2) short-lived behavioural effects after removal of the incentives, and (3) uncertainty about sustainable and equitable funding. Drawing on interdisciplinary evidence, we explore potential solutions such as tailored incentive design, strategies to prolong behavioural change (e.g. intermittent reinforcement or dynamic phase-outs), and the development of public or private funding models. We argue that while financial incentives should not replace structural health policy interventions, they can be a powerful complementary tool. A coordinated research agenda is needed to inform scalable and effective implementation.

## Introduction

In 2019, 18.4% of Europeans aged 15 years and over smoke daily, more than half (i.e. 52%) of Europeans are overweight, with 16% of Europeans classified as obese, and only 12% and 33% of Europeans adhere to recommended guidelines on fruit and vegetables consumption or health-enhancing physical activity [1]. Such unhealthy lifestyles call for the implementation of health interventions that promote healthier lifestyles, ranging from traditional information campaigns or taxation-based interventions, to behavioural interventions [2]. While structural-level health interventions (e.g., those addressing structural determinants of unhealthy behaviours such as poverty or obesogenic environments) are critical in shaping population health, these can be complemented by individual-level interventions that directly target health-related behaviours [3].

One promising individual-level intervention, backed by high-quality evidence showing its effectiveness in many different contexts, is providing financial incentives for healthy behaviours. Systematic reviews and meta-analytic studies, for example, show that financial incentives may be a promising intervention for promoting healthier diets in adults [4–6] and children [7, 8], encouraging smoking cessation [9], particularly during pregnancy [10], and to stimulate physical activity [4, 11, 12]. Financial incentives are used in many different contexts and populations (e.g. with patients in clinical settings or with employees at workplaces), and bibliographic work typically points out that effectiveness of financial incentives may depend on the setting or way in which they are used.^1^

Importantly, financial incentives can potentially reduce widely observed income-related health inequalities (i.e. poorer individuals have lower healthy life expectations, see: [13]), because individuals with lower socio-economic positions (SEP) may respond more strongly to financial incentives [11]. Lower SEP individuals may respond stronger to incentives as for these individuals the same incentive generates more marginal utility (i.e. more strongly increases their purchasing power) compared to individuals with higher SEP [14]. This, together with poorer life expectancy in these groups, may explain why incentives are often used in programs specifically targeted to lower SEP groups (e.g. [15, 16]). There is, however, scant evidence explicitly comparing effectiveness of incentives between SEP groups [17]. While some studies report that the effects of incentives vary by SEP (e.g. [18] for weight loss), other studies find no such evidence (e.g. [19] for COVID vaccinations, or [20] for obesity), suggesting that the degree to which incentives reduce inequalities may depend on context and the incentivized behaviours.

Policymakers, thus, have access to a potentially powerful tool to increase population health and contribute to the reduction of health inequalities. Yet, programs using financial incentives are infrequently used in Europe, and not often applied in large-scale settings (as opposed to the US, where e.g. 81% of large employers provide incentives for health behaviour change, [21]). For example, Dutch health insurers experimented with financial incentives and found encouraging results using a system in which clients earn points through physical activity that can be used to purchase goods, but recently scaled down these initiatives nonetheless [22]. In this perspective, we will explore why health-promoting financial incentives are not yet implemented on a large scale despite their potential. Drawing from the rich, interdisciplinary literature on the use of financial incentives, we provide a working definition of incentives, and identify three challenges, discussed separately, as well as explore their potential solutions and outlining a research agenda.

### Financial incentives: carrots, sticks and not taxes

Most incentive programs can be categorized as belonging to one of two types: carrots or sticks [23]. Carrot financial incentives create opportunity for a gain, e.g. awarding a cash reward for successful behaviour change, or when non-cash rewards are used: electronic devices [24] or a nice holiday [25]. Stick financial incentives promote behaviour change through the threat of a loss. Such stick incentives expose individuals to financial penalties if they are unable to change their unhealthy lifestyles. This is often operationalized by asking individuals to put their own money at stake in a deposit contract, in which they pay an amount upfront that they can earn back through (maintenance of) healthy behaviour (e.g. [26]). Importantly, participants need not always risk losing their own money: financial rewards are often ‘framed’ to be perceived as sticks by assigning an up-front reward that shrinks or is taken away when individuals are unsuccessful in changing their behaviour [27, 28]. Similarly, individuals may be asked to put money they were provided with as part of the financial incentive program at stake in a deposit contract [29]. In this perspective, we will group these stick incentives, considering they both involve the experience of loss, and refer to them as incentives involving (perceived) losses (for an explicit comparison involving incentives with framed and ‘real’ losses, see: [27]).

Financial incentives directly influence individuals’ cost–benefit assessments by offering tangible rewards for engaging in typically unattractive healthy behaviours or by imposing perceived penalties for indulging in unhealthy ones. Unlike taxation-based interventions—which aim to modify behaviour indirectly by embedding costs or subsidies into the market prices of goods (e.g., through 'sin taxes' on sugary or fatty foods or subsidies for healthier alternatives) [2] — financial incentives are linked to the behaviour itself. That is, while taxes are integrated into the product price and influence choices implicitly through market mechanisms, incentives are directly and focally linked to specific individual actions. Moreover, many individuals may suffer from lapses of self-control, rendering them unable to realize planned behaviour change [30]. Such lapses of self-control may result from present bias, i.e. the overweighting of any cost or consequence in the present [31], and cause procrastination. In this context, financial incentives can function as commitment devices, enabling individuals to pre-commit to future healthy actions and follow-up on their planned behaviour change—an effect not typically achieved through taxation [21].

### Financial incentives may not reach those who need them the most

Financial incentives may differ in their attractiveness to potential recipients. Carrot incentive programs in which rewards can be earned can be considered attractive and acceptable (e.g. [32, 33]). Support for financial incentives is far from universal [34], potentially because the use of incentives may be seen as coercive and/or unfair [35], and such lack of support may be more pronounced in some groups and differ between countries (e.g. lower SEP individuals and women, [36],or between the United Kingdom and France, [37]). Beyond issues of acceptability, another challenge lies in ensuring that incentives effectively target those most in need of behavior change. For example, in the context of physical activity, financial incentives interventions have been shown to be most effective for less physically active subgroups [12]. Yet, Dutch health insurer ASR Vitality recently overhauled their incentive program used to promote physical activity because it became too expensive to maintain [38], partially because of high use of the program by individuals who were already physically active. This tendency for groups for whom behaviour change is less needed (e.g. those already engaging in the desired behaviours at baseline) to be overrepresented may be caused by their high confidence in their ability to achieve program goals, but it puts the (cost-)effectiveness of these programs at risk as well as limiting potential effects on population health.

Concerns around not reaching the right populations may even be exacerbated for stick incentive programs, in which individuals are exposed to (perceived) losses. Deposit contracts are a clear example of how stick incentives can be used as a commitment device [21, 39], individuals pay a deposit to commit their future selves to realizing behaviour change goals. These loss-based financial incentive programs are sometimes found to be even more effective than carrots [4, 28, 40], which is explained by individuals’ tendency to assign a larger weight to losses than gains of the same size [41]. Earlier work has shown that deposit contracts are effective in various contexts ranging from weight loss [42, 43], smoking cessation [40, 44] to daily step counts [45, 46, 47], however, deposit contracts only work *for those who are willing and able to pay a deposit.*

Take-up of deposit contract is generally low [40, 45]. Carrera et al. [48] summarize evidence from multiple sources, and find that take up rates range from 11 to 65%, with particularly low take-up of programs in which individuals deposit their own money. Moreover, take-up of deposit contracts appears to be lower in low SEP groups [49]. Given that the unhealthy behaviours that are often the target of financial incentives tend to be more prevalent in low SEP groups in most European countries (e.g. for smoking, see: [50]), the use of stick financial incentives could potentially exacerbate income-related health inequalities. Furthermore, take-up of deposit contracts would likely be lower in individuals who feel they are unlikely to reach the predetermined behaviour change goal. Stick incentives, therefore, raise a risk of excluding vulnerable populations that are most in need of support.

*Solutions & Research agenda*: Tailoring financial incentives such that they reach or reap greater benefit in the targeted (vulnerable) populations may provide a solution [51]. We may distinguish between two complementary approaches: (1) Tailoring the type of incentive offered to match the target population’s characteristics to sort greater effectiveness, and (2) Personalizing the structure or delivery of financial incentives (potentially also relying on individual characteristics). The first approach involves designing financial incentives in a way that they attract or perform particularly well for recipients with specific characteristics. Determining which recipient characteristic is relevant requires a priori behavioural analysis, as this will depend on the behaviour incentivized. For example, earlier work in HIV patients showed that those with stronger risk-seeking tendencies were more likely to benefit from lottery-based incentives [52]. Hence, if one expects that the target population is mostly risk-seeking, it may be worth considering lottery-based incentives. Such lottery-based incentives have been mostly used in US contexts [4], they have also been studied successfully in the European context, e.g. promoting exercise and smoking cessation in the Netherlands [25, 53] and vaccination uptake in Sweden [54].

The second form of tailoring involves personalizing incentives, i.e. offering financial incentive programs in which the structure or delivery of incentives differs between recipients. Examples include: i) allocating rewards based on relative *changes* in behaviour rather than generic goals that are identical for everyone, such that individuals in whom the largest capacity for behaviour change exists (e.g. who are inactive at baseline) receive higher rewards, or other ways of tailoring rewards or goals to individuals’ characteristics [55–57], ii) personalizing the deposit amount in deposit contracts [45] and/or offering a different rate of ‘matching’ (i.e., the program contributes additional funds in proportion to the participant’s deposit) for different individuals, iii) ensuring that rewards align with individuals’ values [51] or what they find valuable (i.e. tickets to football-matches and not the opera), or iv) offering respondents a choice between different incentive schemes, e.g. between piece-rate incentives that reward every occurrence of desired behaviour and ‘streak’ incentives where the reward amount escalates as individuals maintain a continuous streak of desired actions over time [58], or between stick and carrot incentives [59–61]. The (cost-)effectiveness of both types of tailored incentives has, however, not been studied much, and is an important topic for future research.

### Incentives’ effects are often short-lived

For some types of health behaviours, a short-lived behaviour change can suffice to make a meaningful difference in population health. For example, a temporary increase in vaccination uptake in vulnerable populations can reduce the prevalence or impact of communicable disease [62], and temporarily reducing smoking among expecting parents would improve neonatal health even though relapse is likely (for a review, see: [63]). However, sustained behaviour change or inhibition is often needed to reduce the risk of behavioural health problems. Unfortunately, meta-analyses show that, depending on the targeted behaviours, incentive effects typically decline 3 months after incentive removal, with sporadic evidence of sustained effects [5, 11, 17, 64]. This lack of sustained effects is a key challenge preventing wider use financial incentives, as those hoping to use financial incentives to promote lasting healthier habits will usually be disappointed: long-term behaviour change requires long term behavioural support.

*Solutions & Research agenda*: A straightforward solution to encourage the use of financial incentives would be to focus or restrict their use to behaviours for which short-lived changes suffice. For example, financial incentives have been successfully used to increase the uptake of COVID-vaccines in various countries [19, 65, 66], without any evidence for negative spillover effects that are often a source of concern [67]. Similar high-quality evidence exists for the use of financial incentives to promote smoking cessation in expectant parents *during pregnancy* [68–70]. If, on the other hand, sustained behaviour change is required, we should consider developing financial incentive programs that can be offered in the long-term.

If incentives cannot be offered long-term due to lack of sustained funding, alternative incentive schemes could be explored. For example, it has been shown that when financial incentives were provided on intermittent reinforcement schemes rather than fixed per visit payments, they were more effective in facilitating long term maintenance gym visits [71]. Future research could design dynamic financial incentives that gradually fade out while habits develop [72], and measure their impact on long term maintenance of behaviour change. Such programs could benefit from monitoring individuals’ habit formation to decide if incentives can be phased out in these groups, or identifying those groups or individuals who need support on the longer-term. Behavioural scientists can support this by focusing research efforts on the comparative long-term effectiveness of different types of financial incentives, as well as studying how to best phase out incentives (e.g. is it better to decrease the size or frequency of incentives?).

### Prevention suffers from uncertain funding

Currently, experimentation with financial incentives is often occurring within workplaces, clinical settings or academic institutions, as part of efforts to better understand their effects [4, 73]. Usually, such projects are funded temporarily, and no, or only limited funding is available for large scale implementation. When considering scaling up financial incentive programs it is crucial to consider who should provide the funding: e.g. the participants of the program (through deposit contracts), their employers, health insurers, or other public bodies.

Finding sustainable funding and the right funder to provide it is complex. This point is evidenced by the scaling down of Dutch health insurers large-scale financial incentive programs. Dutch health insurers indicated that the costs of the program were higher than anticipated and benefits were uncertain [38]. Indeed, the benefits of financial incentives are predominantly experienced by the individuals participating in these programs (improved health and wellbeing), whereas indirect benefits to employers (reduced absenteeism) or insurers (decreased health expenditures) may occur when recipients of incentives have switched jobs or insurers (or not at all).

From a societal perspective, the use of financial incentives can be cost-effective (e.g. for smoking, see: [74]), but funding these incentives through public expenditures may lead to resistance from taxpayers, policy-makers or healthcare professionals. In particular, some find the provision of financial rewards for behaviour (change) that others have engaged in without the presence of reward unfair [35]. Large scale funding of financial incentives through public expenditure is also contraindicated by the apparent reduction in solidarity towards individuals with unhealthier lifestyles. For example, Dutch evidence shows a declining willingness among the public to collectively bear healthcare costs resulting from unhealthy lifestyles [75]. Across many European countries, there is also growing support for factoring lifestyle choices into decisions about healthcare prioritization and cost-sharing expectations, particularly for individuals engaging in unhealthy behaviours [76]. However, although their annual healthcare expenditures are higher, healthcare expenditures for those with unhealthy lifestyles are typically *lower* than for those with healthy lifestyles when taking a *lifetime perspective* [77, 78], due to their reduced lifespan. This will be particularly true in countries such as the Netherlands and Scandinavian countries in which long-term care expenditures (e.g. for the elderly) are relatively high [79], as unhealthy lifestyles will prevent a proportion of individuals from requiring age-related long-term care. Hence, providing financial incentives that promote behaviour change in those with unhealthier lifestyles may not be unfair at all – as this could reduce inequality in lifetime healthcare expenditures.

*Solutions & Research agenda*: Many European countries face challenges in finding sustainable funding structures for preventative interventions [80], such as financial incentives. In the Netherlands, the government was recently advised to apply the same decision and reimbursement framework to curative, long-term and preventative interventions [81]: to align with the principle that all health gains are treated equally regardless of how and in whom they are generated [82, 83]. For financial incentives this would imply that – similar to funding of medical treatments – they are funded through public expenditures if they are found to be cost-effective in economic evaluations. This implies a radical change in perspective: financial incentives no longer need to save costs (which is the expectation some have for preventative measures, as discussed by: [84]). Instead, the provision of financial incentives is seen as desirable when they generate sufficiently valuable health and societal benefits to warrant their price, compared to some agreed-upon threshold. The Dutch committee advising on costs and benefits of prevention recommend using a single threshold across all health-generating activities [81]. When considering health systems recommending economic evaluation with a healthcare perspective, i.e. using thresholds based on the opportunity costs of healthcare spending (e.g. the United Kingdom, see: [85]), it is not clear a priori how to deal with thresholds for prevention (as this typically occurs outside of the formal healthcare sector), which may require redefining the scope of opportunity costs.

Regardless, such a paradigm shift necessitates sufficient public solidarity: it hinges on taxpayers’, policy-makers’ and healthcare professionals’ acceptance of funding prevention (e.g. financial incentives) in vulnerable populations in which unhealthy lifestyles are predominant by. A potentially less challenging solution would be for individual consumers or employers to fund financial incentives (following the example of many employers in the United States, [21], and recently the Netherlands, [25]). Individuals and employers may rely on commercial parties to develop and implement financial incentive programs, such as the examples of companies like Waybetter (in the U.S and worldwide) or European alternatives such as Fitcoin (in the Netherlands), WeWard (in France), or BeatTheStreet (in the United Kingdom). Participating in incentive programs run by these for-profit organizations that charge individual consumers or employers for participation has been found to be effective in promoting healthier behavior [86]. If no external funding is available, deposit contracts could be a solution, which can in principle be completely funded by individual consumers. However, relegating funding of incentives to employers or individual consumers implies that only those (with employers) with sufficient willingness to pay get access to this powerful tool for health promotion, and vulnerable populations (i.e. unemployed or low SEP) in which benefits may be largest could miss out.

## In conclusion

Financial incentives can contribute to preventing lifestyle-related morbidity and mortality. We encourage scaling up the use of financial incentives, particularly to encourage behaviour change in contexts where high-quality evidence supports their use, and/or public acceptability exists. For example, high-quality evidence supports the use of incentives in smoking cessation [9, 10] and some public support exists for this type of program in vulnerable populations [35] and during pregnancy [36, 37] – suggesting that this is a context in which scaling up of financial incentive programs is particularly promising. However, the use of incentives should complement and by no means replace policy targeting structural determinants of unhealthy behaviour, such as policies addressing obesogenic environments, poverty, or national/international laws and regulation. Indeed, health behaviour is a consequence of individual and such contextual influences [87]. Scaling up programs involving financial incentives may cause attitudes to shift to individual responsibility, framing lifestyle as in an individual choice rather than a consequence of a complex interaction that also involves powerful structural influences outside the individual [3]. Widespread use of financial incentives would, thus, be desirable, if and only if this coincides with attention to and policy targeting structural and environmental influences of health behaviour.

Of crucial importance in paving the way for widespread use of financial incentives is applied policy-relevant research addressing the challenges we covered in this perspective: reaching the right groups through tailoring, better understanding how to reach long-term effectiveness of incentives, sustainable funding models and acceptability of financial incentives. Indeed, future research should go beyond showing that incentives work, and instead focus on *how, why* and *under which conditions* they work—by identifying underlying behavioural mechanisms and linking them to scalable designs that practitioners and policymakers can adapt to local contexts. In our contribution, we have relied mostly on examples from the Netherlands and the United states. To further the field, we invite policy makers and researchers across Europe to join us in exploring ways forward towards large scale implementation of health promoting financial incentives.

## Footnotes

1 Substantial heterogeneity in effectiveness of financial incentives is, for example, reported or discussed in each the following bibliographic studies cited in our perspective: Kurti et al. [5],Giles et al. [17],Notley et al. [9],Boonmanunt et al. [4],Purnell et al. [6],and Mitchell et al. [12]. Note that some cited bibliographic studies do not find evidence for heterogeneity related to the operationalization of incentives (or do not discuss it): Mantzari et al. [11],Belot and James [7],Jensen et al. [8],Tappin et al. [10].
